# Evaluating motion of pancreatic tumors and anatomical surrogates using cine MRI in 0.35T MRgRT under free breathing conditions

**DOI:** 10.1002/acm2.13930

**Published:** 2023-04-18

**Authors:** Benjamin Lewis, Anamaria Guta, Jaeik Shin, Zhen Ji, Jin Sung Kim, Taeho Kim

**Affiliations:** ^1^ Department of Radiation Oncology Washington University School of Medicine St. Louis Missouri USA; ^2^ Department of Radiation Physics The University of Texas MD Anderson Cancer Center Houston Texas USA; ^3^ Department of Radiation Oncology Yonsei University College of Medicine Seoul Republic of Korea

**Keywords:** cine MRI, MRgRT, pancreatic tumor, target tracking

## Abstract

Treatment tolerability is a significant limitation to pancreatic cancer treatment with radiotherapy due to proximity to highly radiosensitive organs and respiratory motion necessitating expanded target margins. Further, pancreatic tumors are difficult to visualize on conventional radiotherapy systems. Surrogates are often used to locate the tumor but are often inconsistent and do not provide strong positional relations throughout the respiratory cycle. This work utilizes a retrospective dataset of 45 pancreatic cancer patients treated on an MR‐Linac system with cine MRI acquired for real‐time target tracking. We investigated intra‐fraction motion of tumors and two abdominal surrogates, leading to prediction models between the tumor and surrogate. Patient specific motion evaluation and prediction models were generated from 225 cine MRI series acquired during treatment. Tumor contours were used to evaluate the pancreatic tumor motion. Linear regression and principal component analysis (PCA) based models were used to predict tumor position from the anterior‐posterior (AP) motion of the abdominal surface, the superior‐inferior (SI) motion of the diaphragm, or a combination. Models were evaluated using mean squared error (MSE) and mean absolute error (MAE). Contour analysis showed the average pancreatic tumor motion range was 7.4 ± 2.7 mm and 14.9 ± 5.8 mm in the AP and SI directions, respectively. The PCA model had MSE of 1.4 mm^2^ and 0.6 mm^2^, for the SI and AP directions, respectively, with both surrogates as inputs for the models. When only the abdomen surrogate was used, MSE was 1.3 mm^2^ and 0.4 mm^2^ in the SI and AP directions, while it was 0.4 mm^2^ and 1.3 mm^2^ when only the diaphragm surrogate was used. We evaluated intra‐fraction pancreatic tumor motion and demonstrated prediction models between the tumor and surrogate. The models calculated the pancreatic tumor position from diaphragm, abdominal, or both contours within standard pancreatic cancer target margin, and the process could be applied to other disease sites in the abdominothoracic cavity.

## 1 INTRODUCTION

Target positioning is an essential step for delivering high quality and effective treatment of cancer in radiotherapy. Small variations, on the order of 5 mm, can result in geometric miss of the target and irradiating healthy tissue. Extensive work has been applied to accurately position patients using immobilization devices, laser alignment systems, and on‐board imaging devices.^1^, ^2^ However, even the most painstaking setup measures can be defeated by a moving target.^3^, ^4^ A common source of motion in radiotherapy is respiration, which can result in target displacements of up to 50 mm, especially in the abdominothoracic region.^5^, ^6^, ^7^ Tumors near the diaphragm experience the greatest extent of this semi‐periodic motion, with few bony structures nearby to use as surrogates in X‐ray imaging. To improve treatment of these tumors, gating methods have been implemented to only deliver radiation when the tumor, or a surrogate for the tumor, is in a specified position.^8^, ^9^, ^10^ These methods often introduce their own complications such as uncertainty in the link between external surrogates and the tumor, increased treatment delivery time, patient discomfort, and dedicated treatment machines that are unobtainable for many radiotherapy departments.

Accounting for tumor motion is especially important for targets that respond best to escalated doses and hypo‐fractionated treatment, due to the risk of depositing a large percentage of the prescription dose to surrounding healthy tissue. One such disease type is pancreatic cancer, which is in close proximity to multiple organs at risk (OARs), and treatment tolerability is often a limiting factor to deliverable dose to the target.^11^, ^12^, ^13^ In this case, MR‐guided radiotherapy (MRgRT) has become one of the most desirable treatment modalities due to imaging with excellent soft tissue contrast and real‐time tumor tracking. Recent studies have shown that MRgRT can provide excellent local control of disease with low rates of normal tissue toxicity.^14^, ^15^, ^16^ However, the cost of establishing and maintaining an MRgRT program is extremely expensive and not feasible for the majority of radiation oncology departments. However, understanding patient specific respiratory motion using real‐time imaging methods such as cine MRI could help improve motion management and treatment delivery methods using conventional treatment systems.

MRgRT with real time tumor tracking can provide a valuable data source for accomplishing this task. During treatment delivery, patients undergo 2D sagittal cine MRI with active tumor tracking for beam gating. Our institution has collected a unique dataset of multi‐fraction cine MRI for 45 pancreatic cancer patients, which includes the abdomen and diaphragm within the field of view (FOV), as well as tumor tracking contours.

In this study we analyzed pancreatic tumor and surrogate motion during free breathing for 45 patients with five treatment fractions each, for a total of 225 MRgRT treatment fractions, then applied multiple tumor position prediction models to assess the accuracy of intra‐fraction position estimates based on common tumor surrogates.

## 2 METHODS

This study includes 45 retrospectively selected pancreatic cancer patients treated at our institution. All patients underwent five fractions of MRgRT, with a prescription dose of 50 Gy, on a 0.35T ViewRay MRIdian MR‐Linac system (ViewRay, Oakwood Village, OH). The ViewRay MRIdian system incorporates a 0.35T split bore superconducting magnet with a circular gantry assembly, including a linac that generates a 6 MV flattening filter free photon beam with a dose rate of 600 cGy/min at a source‐to‐axis distance of 90 cm, placed within the gap between the magnets, it has been previously described in detail.^17^ The clinical workflow for patients treated on the MR‐Linac system has been previously described by Fischer‐Valuck et al.^18^ For target delineation, the original GTV was generated from registered MR and CT images (MR fusion) and drawn by a physician. On the day of treatment, a volumetric MRI was acquired, and planning contours were transferred to the daily MRI. The treating physician would then manually modify contours including the original GTV as necessary, and the plan recalculated on the daily anatomy. The patients in this study were treated under free breathing conditions with beam gating based on real time cine‐MRI acquisition and real time target segmentation by the ViewRay system. The cine images were continuously acquired so they represent a complete record of motion in two dimensions. The real time tumor tracking using sagittal 2D cine MRI acquired at four frames per second (FPS) during free breathing. The sagittal 2D cine imaging parameters were TR/TR = 2.1/0.91 ms, flip angle = 60°, rBW = 1351 Hz/pixel, FOV = 350 × 350 × 7 mm^3^, and imaging matrix = 100 × 100 × 1. All clinical images were then automatically interpolated by the ViewRay treatment delivery system (TDS) to a matrix size of 466 × 466 × 1 pixels, with a pixel size of 0.75 × 0.75 mm^2^. The first 1000 imaging frames from each cine MRI acquisition were used, for a total of 214,559 images from 225 fractions. 1000 imaging frames were used due to empirical reasons such as the time required to create and examine contours on each frame. For the five fractions displaying the 1st, 25th, 50th, 75th, and 100th percentile superior‐inferior (SI) tumor motion range, all frames were used, ranging from 2981 to 6331 frames per fraction with the initial 700 frames used for training.

### 2.1 Region delineation and motion quantification

The treatment target was delineated automatically by the ViewRay system in real time as images were acquired during treatment, which included a small area of soft tissue surrounding the tumor. After treatment was completed, cine MRI images with embedded contours were exported from the treatment system. An in‐house software titled MAXgRT, developed in MATLAB 2020B (Mathworks, Natick, MA), was then used to extract contours and semi‐automatic contouring of the diaphragm and the anterior abdominal surface. The embedded contours were considered to be the clinical contours, and the off‐line contours generated during data processing were considered to be the in‐house contours.

Due to the additional tissue included in the clinical tumor contour, an in‐house tumor contour was generated by automatically deforming the clinical tumor contour to reduce surrounding tissue and generate a tighter in‐house tumor contour. This was done so that the center of mass calculation for the target position was more accurate. The active contouring, or snakes, method was used to delineate the tumor region based on the original tumor contour. This method was based on the Chan‐Vese model, which is able to implement segmentation of objects without clearly defined boundaries in images that would not be suitable to segmentation by thresholding or gradient based methods.^19^ The Chan‐Vese model attempts to iteratively minimize an energy function with weighted values corresponding to the sum of intensity differences from the average values inside and outside the contour region, and a term dependent on the length of the contour boundary. The clinical tumor contour from the ViewRay system and the in‐house tumor contour were used to generate two center of mass coordinates on each cine MRI frame, which served as the tumor position coordinate.

After automatic generation of the in‐house contour, MAXgRT provided semi‐automatic contouring of the diaphragm and anterior abdominal surface. For diaphragm contouring, the MAXgRT software requires an initial user designated contour. The snakes algorithm is applied to the user contour on the initial frame, and each subsequent frame uses the prior frame's contour as the starting point for the snakes algorithm. After the contours were generated, any mismatches were manually corrected slice‐by‐slice. Each column containing the diaphragm contour served as a sample for the SI motion direction.

**FIGURE 1:**
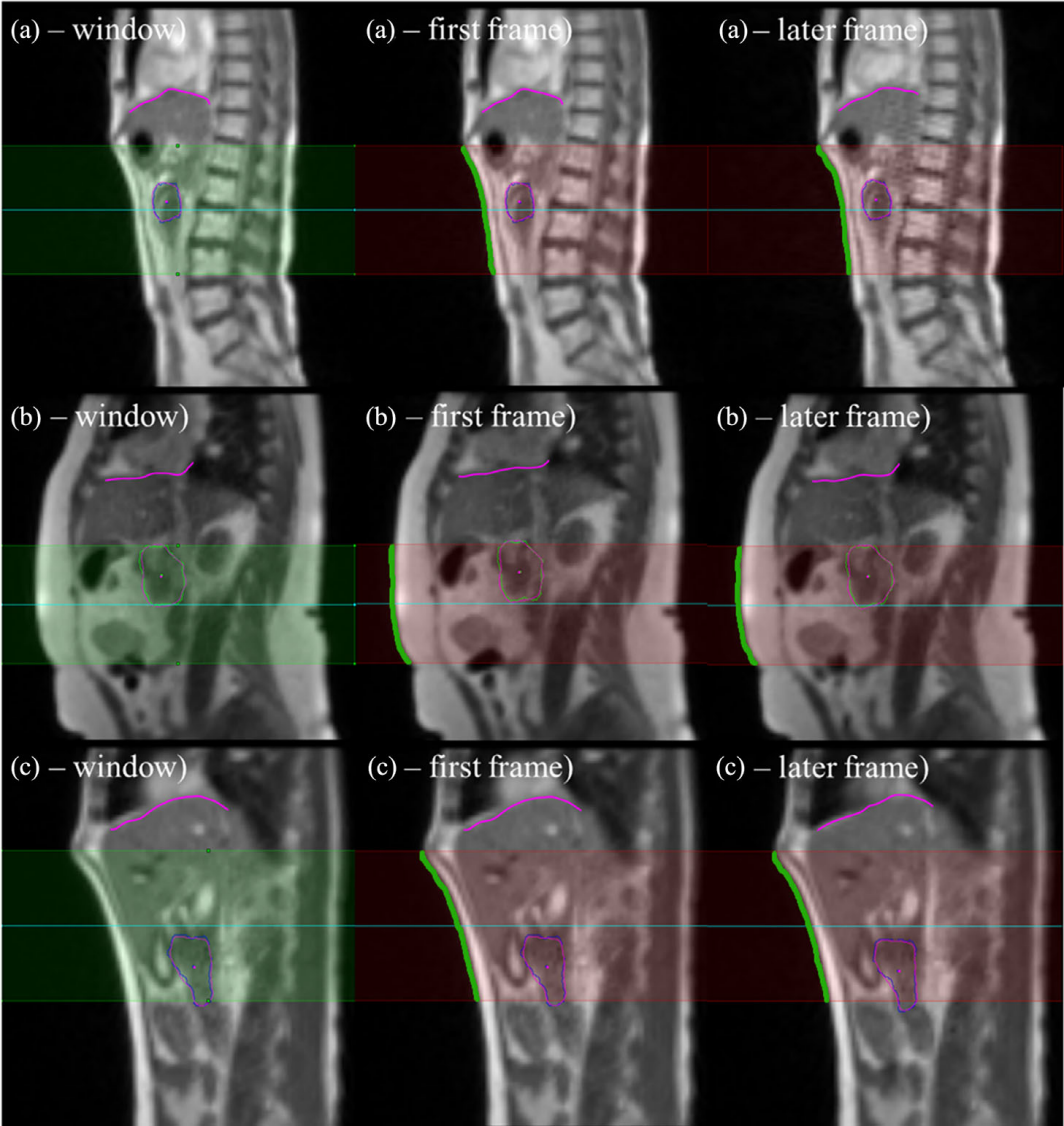
Example images from the MAXgRT software of the abdominal contour window (window), generated contour for the first frame (first frame), and the generated contour for a random slice (later frame). Each row indicates a different patient from this study. The green line indicates the abdominal contour, and the cyan line indicates the diaphragm contour.

The abdominal surface contour was generated by MAXgRT from a user defined boundary region. The boundary region was set to extend 12 cm inferiorly from the base of the rib cage, which encompassed the region of greatest motion. The snakes algorithm was again used to detect the boundary between abdominal surface and the image background. Similar to the diaphragm contour, each row of the abdominal surface contour was used as a sample for the anterior‐posterior (AP) motion direction. The abdominal contour creation window and example contours are shown in Figure 1.

### 2.2 Tumor position prediction models

Tumor position prediction was performed using contour information from (1) only the abdomen AP position, (2) only the diaphragm SI position, and (3) the combination of abdomen SI position with diaphragm AP position. Two modeling approaches were applied for each surrogate combination. Model 1 was a ridge regression model based on the relationship between tumor center and the surrogates from the same frame. The linear models for X (AP) and Y (SI) position prediction and objective functions with L‐2 norm regularization are described by Equation 1.

(1) $$\begin{array}{ccc} X_{t,p} & = & a+\sum_{i=1}^{n}w_{d,i}X_{d,i}+\sum_{l=1}^{n}w_{a,l}Y_{a,l}\;with\\ & & \times \;\sum_{1}^{N}{\left(X_{t}-\left(a+\sum_{i=1}^{n}w_{d,i}X_{d,i}+\sum_{l=1}^{n}w_{a,l}Y_{a,l}\right)\right)}^{2}\\ & & +\;\sum_{i=1}^{n}w_{d,i}+\sum_{l=1}^{n}w_{a,l}<\varepsilon \\ Y_{t,p} & = & a+\sum_{i=1}^{n}w_{d,i}X_{d,i}+\sum_{l=1}^{n}w_{a,l}Y_{a,l}\;with\\ & & \times \;\sum_{1}^{N}{\left(Y_{t}-\left(a+\sum_{i=1}^{n}w_{d,i}X_{d,i}+\sum_{l=1}^{n}w_{a,l}Y_{a,l}\right)\right)}^{2}\\ & & +\;\sum_{i=1}^{n}w_{d,i}+\sum_{l=1}^{n}w_{a,l}<\varepsilon \end{array}$$

Where N is the number of images, n is the number of image pixels along the X or Y dimension, $X_{t}$ and $Y_{t}$ are the true X and Y position of the tumor center, $X_{t,p}$ and $Y_{t,p}$ are the predicted AP and SI position of the tumor center, $X_{d,i}$ is the X position on the Y projection at the i‐th pixel from the abdomen contour, $Y_{a,l}$ is the Y position on the X projection at the l‐th pixel from diaphragm contour, $w_{d,i}$ and $w_{a,l}$ are weighting factors for each pixel, and ε=0.01. For diaphragm only modeling all abdomen coefficients were set to 0 and for abdomen only modeling all diaphragm coefficients were set to 0.

Model 2 utilized principal component analysis (PCA) to optimize the dimensions of each feature. The linear models on principle component space for AP and SI position prediction and objective functions with L‐2 norm regularization are described by Equation 2.

(2) $$\begin{array}{ccc} & & P_{x}\left(P_{x,1},P_{x,2},\text{…},P_{x,R}\right)\\ & & \;=PCA\left(\begin{array}{cccc} X_{t,1} & X_{d11},Y_{a11} & \cdots & X_{d1,n},Y_{a1,n}\\ \vdots & \vdots & \ddots & \vdots \\ X_{t,N} & X_{dN1},Y_{aN1} & \cdots & X_{dN,n},Y_{aN,n} \end{array}\right)\\ & & X_{t,p}=a+\sum_{i=1}^{R}w_{i}P_{x,i}\;with\\ & & \sum_{1}^{N}{\left(X_{t}-\left(a+\sum_{l=1}^{R_{95}}w_{i}P_{x,i}\right)\right)}^{2}+\sum_{i=1}^{R_{95}}w_{i}<\varepsilon \\ & & P_{y}\left(P_{y,1},P_{y,2},\text{…},P_{y,R}\right)\\ & & \;=PCA\left(\begin{array}{cccc} Y_{t,1} & X_{d11},Y_{a11} & \cdots & X_{d1,n},Y_{a1,n}\\ \vdots & \vdots & \ddots & \vdots \\ Y_{t,N} & X_{dN1},Y_{aN1} & \cdots & X_{dN,n},Y_{aN,n} \end{array}\right)\\ & & Y_{t,p}=a+\sum_{i=1}^{R}w_{i}P_{y,i}\;with\;\sum_{1}^{N}{\left(Y_{t}-\left(a+\sum_{l=1}^{R_{95}}w_{i}P_{y,i}\right)\right)}^{2}\\ & & \;\;+\;\sum_{i=1}^{R_{95}}w_{i}<\varepsilon \end{array}$$

Where $P_{x}$ and $P_{y}$ are the principal components from PCA, *R*_95_ are the components taken with a cumulative explained variance over 95%, and ε=0.01. The intra‐fraction models were trained using 70% of the frames from each fraction. For five patients, an entire fraction was analyzed using the first 700 frames for training and the remainder for testing.

The model predictions were then evaluated using mean squared error (MSE) described by Equation 3 and mean absolute error (MAE) described by Equation 4.

(3) $$\begin{array}{ccc} MSE & = & \frac{1}{N\;samples}\sum^{N\;samples}\\ & & \times \;{\left(Prediction-Truth\right)}^{2}\left[mm^{2}\right] \end{array}$$

(4) $$\begin{array}{ccc} MAE & = & \frac{1}{N\;samples}\sum^{N\;samples}\\ & & \times \;\left|Prediction-Truth\right|\left[mm\right] \end{array}$$

These metrics were applied to the AP and SI directions separately.

## 3 RESULTS

### 3.1 Motion range

#### 3.1.1 Pancreatic tumor motion

**FIGURE 2:**
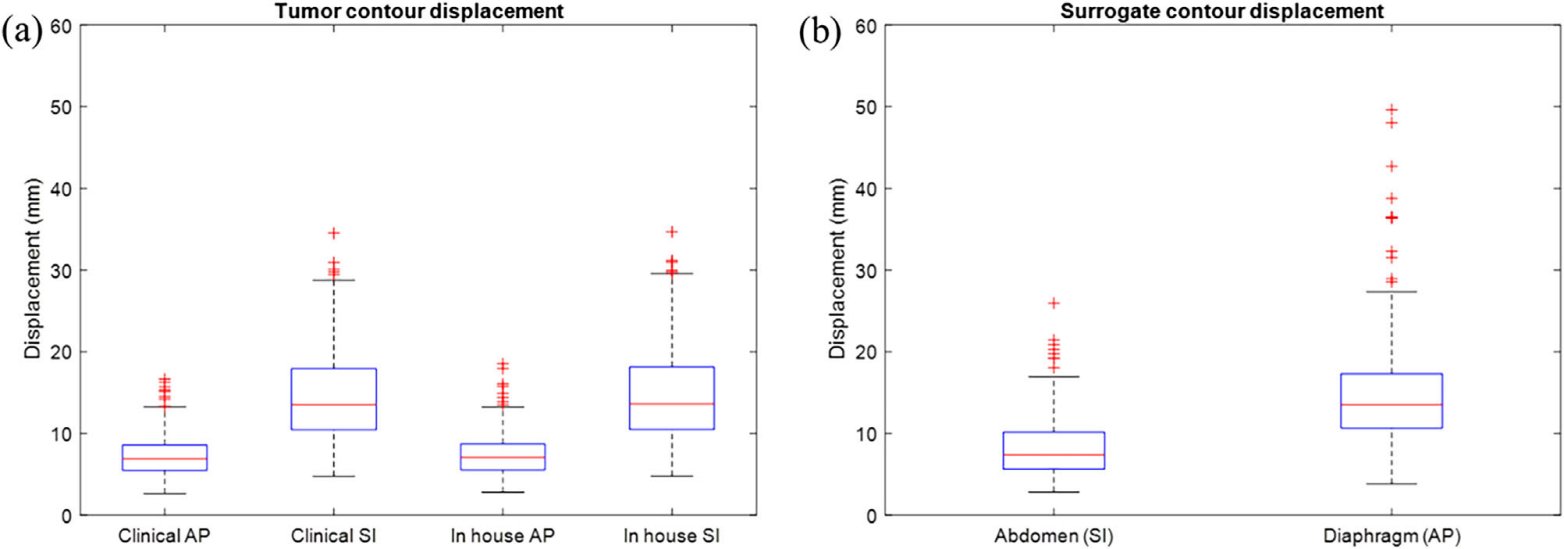
(a) The tumor contour displacement in the anterior‐posterior (AP) and superior‐inferior (SI) directions for the clinical and in‐house tumor contours. (b) The diaphragm and abdomen contour displacements in the AP and SI directions, respectively. For both plots, the bottom and top edge of the box indicate the 25^th^ and 75^th^ percentile, respectively, outlier data points are indicated with the + symbol.

Pancreatic tumor motion range was similar between the clinical and in‐house contours. The clinical contour had an average displacement of 7.3 ± 2.6 mm in the AP direction, and 14.7 ± 5.7 mm in the SI direction, while the in‐house contour showed a displacement of 7.4 ± 2.7 mm and 14.9 ± 5.8 mm in the AP and SI directions, respectively. Figure 2a shows the extent of tumor displacement in the AP and SI directions for both the clinical and in‐house tumor contours.

**FIGURE 3:**
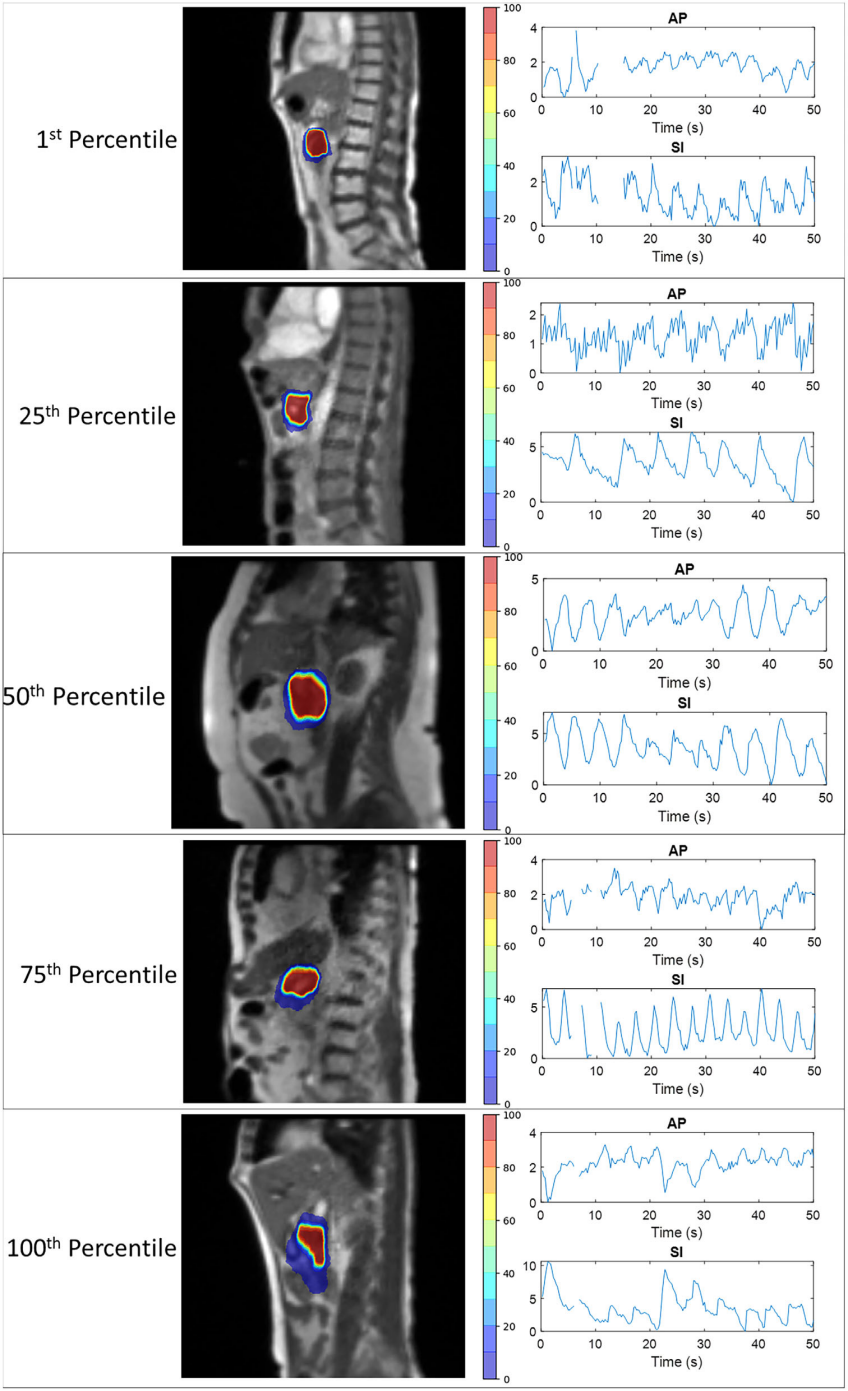
Intra‐fraction variation of pancreatic tumor contour position normalized to the number of total acquired frames for five patients representing the 1^st^, 25^th^, 50^th^, 75^th^, and 100^th^ percentile motion range in the superior‐inferior (SI) direction, as well as the paired anterior‐posterior (AP) and SI motion trace over the first two hundred cine MRI frames. Regions of motion plots that are empty indicate that the treatment delivery system was unable to track the target at that point in time.

Individual patients had a significant range in tumor motion, with a minimum motion of 2.8 mm to a maximum of 18.5 mm in the AP direction, and from 4.8 mm to 34.7 mm in the SI direction. Figure 3 shows a heat map of the accumulated contour positions from each cine MRI frame for five patients, which represent the 1^st^ (least), 25^th^, 50^th^, 75^th^, and 100^th^ (greatest) percentile motion range in the SI direction, as well as the paired SI motion trace over 100 cine MRI frames. The gaps in the tumor motion trace indicate regions where TDS did not generate a tumor contour, this generally occurs during gantry motion.

The difference in area between the clinical and in‐house contours was compared for five non‐sequential patients, a total of 25 fractions. In‐house contours were 2.73% ± 0.06% larger, on average, than the clinical contours. This resulted in the centroid position changing by a half pixel length.

#### 3.1.2 Surrogate motion

**FIGURE 4:**
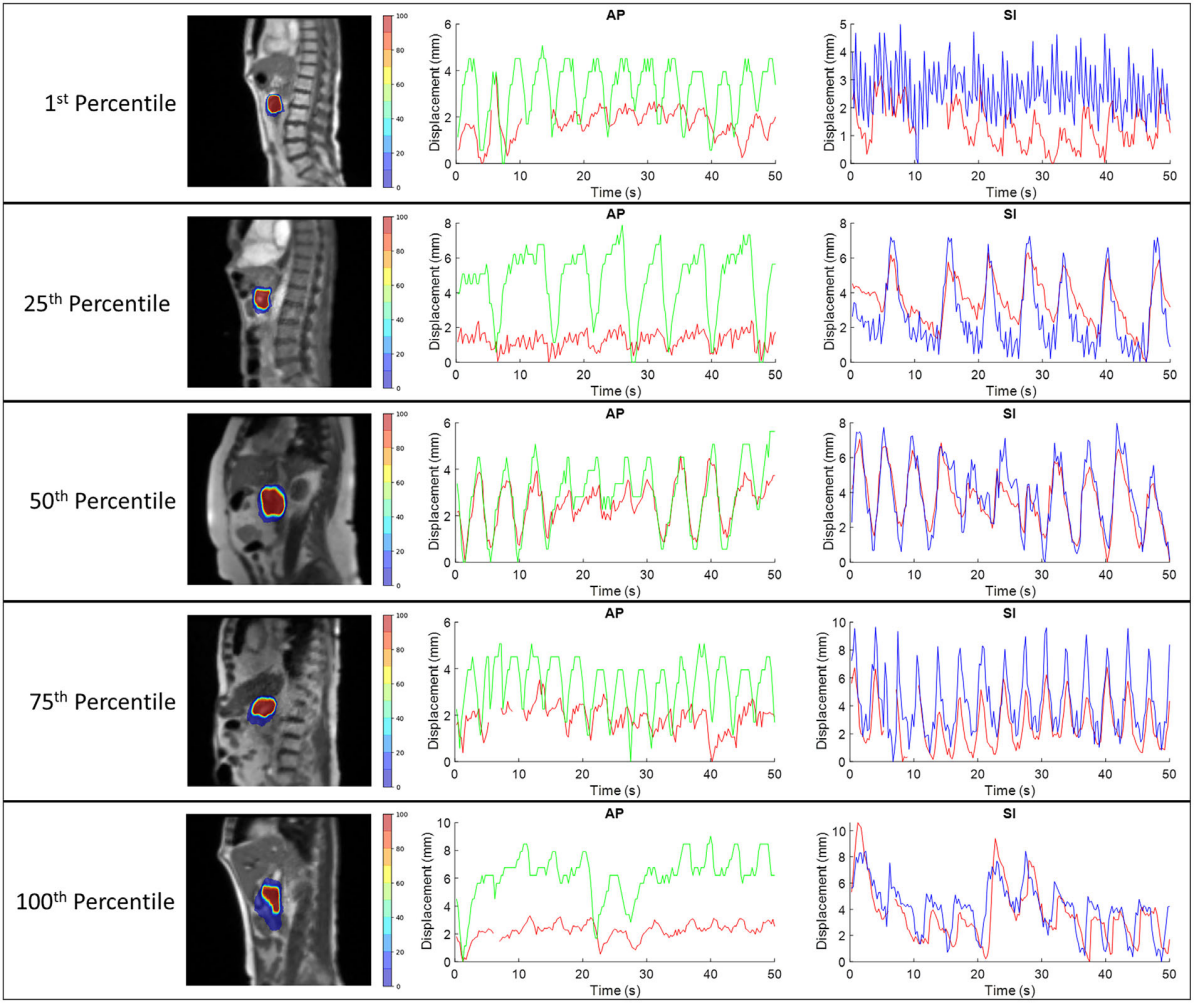
Pancreatic tumor and surrogate contour motion in the anterior‐posterior (AP) and superior‐inferior (SI) directions for five patients representing the 1^st^, 25^th^, 50^th^, 75^th^, and 100^th^ percentile tumor motion range in the SI direction. The red line represents the tumor position in both AP and SI graphs. The green line represents abdomen motion, and the blue line represents the diaphragm motion. The blue and green arrows indicate the position of the abdomen and diaphragm contours, respectively.

The abdomen line contour had a motion range of 2.8 mm to a maximum displacement of 25.9 mm in the AP direction, and the diaphragm line contour had a motion range of 3.8 mm to a maximum displacement of 49.7 mm in the SI direction. Figure 2b shows the extent of diaphragm and abdomen motion, including outliers. Figure 4 shows a comparison of surrogate and pancreatic tumor contour motion in the AP and SI directions for the same five patients shown above.

### 3.2 Intra‐fraction tumor position prediction error

#### 3.2.1 Tumor position prediction error using abdomen surrogate

**FIGURE 5:**
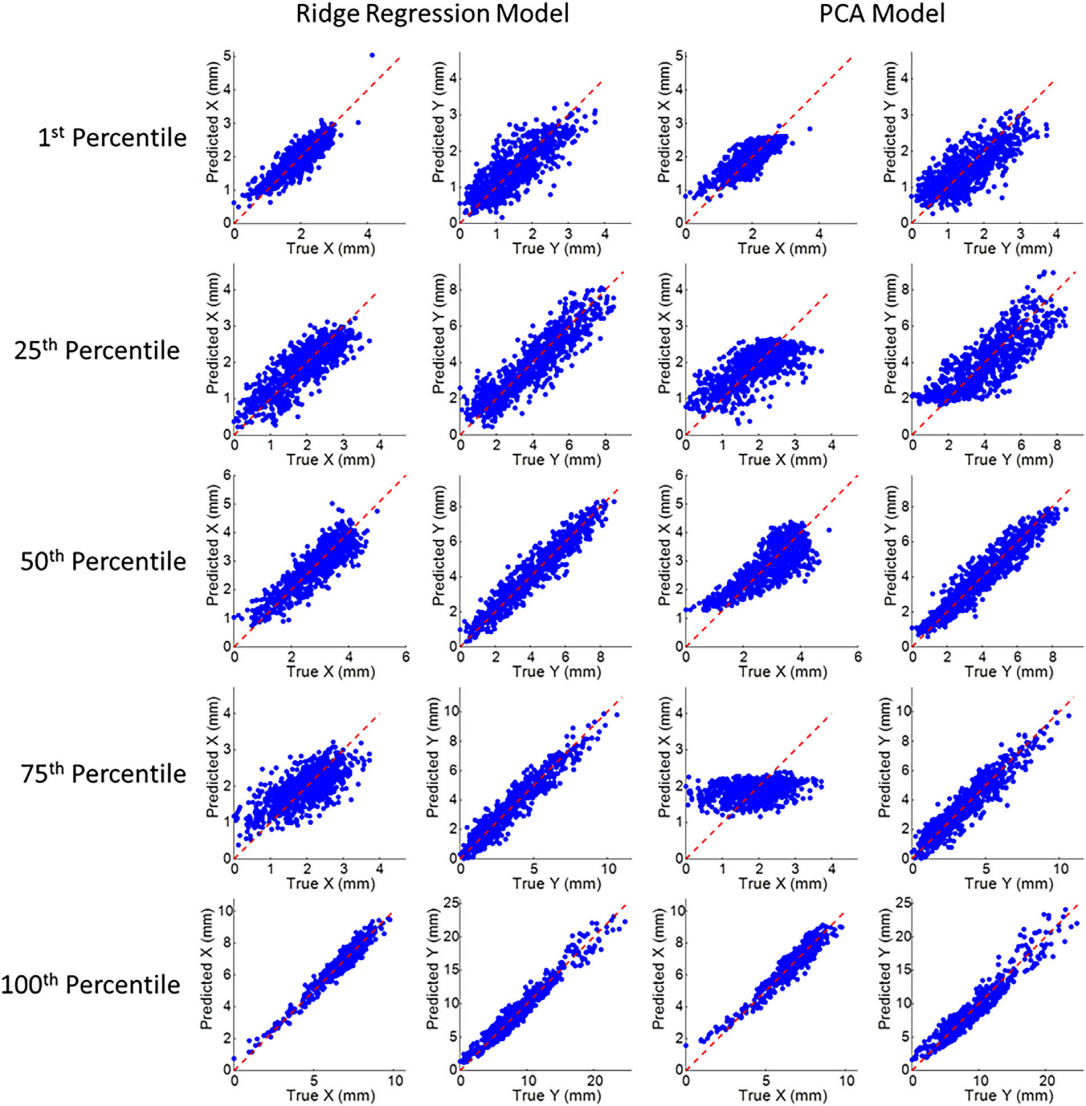
Comparison of true and predicted tumor position from abdomen surrogate based intra‐fraction models using the ridge regression and principal component analysis methods, in the X or anterior‐posterior (AP) and the Y or superior‐inferior (SI) directions. The dashed red line represents unity. Each row corresponds to the five patients representing the 1^st^, 25^th^, 50^th^, 75^th^, and 100^th^ percentile tumor motion range in the SI direction

Table 1 displays the MSE and MAE of intra‐fraction pancreatic tumor position prediction using only the abdomen contour surrogate. The 95^th^ percentile (p95) MSE was 1.2 mm^2^ (max.: 4.4 mm^2^) and 4.3 mm^2^ (max.: 11.2 mm^2^) in the AP and SI directions for the ridge regression model, while the PCA model had a p95 MSE of 1.1 mm^2^ (max.: 3.8 mm^2^) and 3.6 mm^2^ (max.: 12.3 mm^2^) in the AP and SI directions. The ridge regression model also had a p95 MAE of 0.9 mm (max.: 1.6 mm) and 1.5 mm (max.: 2.6 mm) in the AP and SI directions, with the PCA model having values of 0.8 mm (max.: 1.4 mm) and 1.4 mm (max.: 2.7 mm). Figure 5 plots true versus predicted tumor position for the same five patients as prior figures.

**TABLE 1:** The mean (±1SD) of mean squared error (MSE) and mean absolute error (MAE) for intra‐fraction tumor position prediction models using 70% training images and applied to the remaining 30% of frames in the anterior‐posterior (AP) and superior‐inferior (SI) directions, with the clinical tumor contour

		AP		SI	
		MSE (mm^2^)	MAE (mm)	MSE (mm^2^)	MAE (mm)
Ridge‐Clinical Contour	Abd	0.4 ± 0.4	0.5 ± 0.2	1.2 ± 1.3	0.8 ± 0.3
	Dia	0.4 ± 0.5	0.4 ± 0.2	1.1 ± 1.7	0.7 ± 0.3
	Abd+Dia	0.5 ± 0.6	0.5 ± 0.2	1.8 ± 5.4	0.9 ± 0.4
Ridge—In‐house contour	Abd	0.5 ± 0.4	0.5 ± 0.2	1.3 ± 1.5	0.8 ± 0.4
	Dia	0.4 ± 0.5	0.5 ± 0.2	1.1 ± 1.6	0.7 ± 0.3
	Abd+Dia	0.6 ± 0.9	0.6 ± 0.3	1.8 ± 5.4	0.9 ± 0.5
PCA—Clinical contour	Abd	0.4 ± 0.3	0.∖4 ± 0.2	1.3 ± 1.5	0.8 ± 0.3
	Dia	0.4 ± 0.4	0.5 ± 0.2	1.3 ± 1.5	0.8 ± 0.4
	Abd+Dia	0.6 ± 0.6	0.6 ± 0.3	1.3 ± 1.5	0.8 ± 0.4
PCA—in‐house contour	Abd	0.4 ± 0.4	0.5 ± 0.2	1.3 ± 1.5	0.8 ± 0.4
	Dia	0.4 ± 0.4	0.5 ± 0.2	1.3 ± 1.6	0.8 ± 0.4
	Abd+Dia	0.6 ± 0.7	0.6 ± 0.3	1.4 ± 1.7	0.8 ± 0.4

Abbreviations: Abd, abdominal contour‐based model; Dia, diaphragm contour‐based model; Abd+Dia, abdominal and diaphragm contour‐based model.

#### 3.2.2 Tumor position prediction error using diaphragm surrogate

**FIGURE 6:**
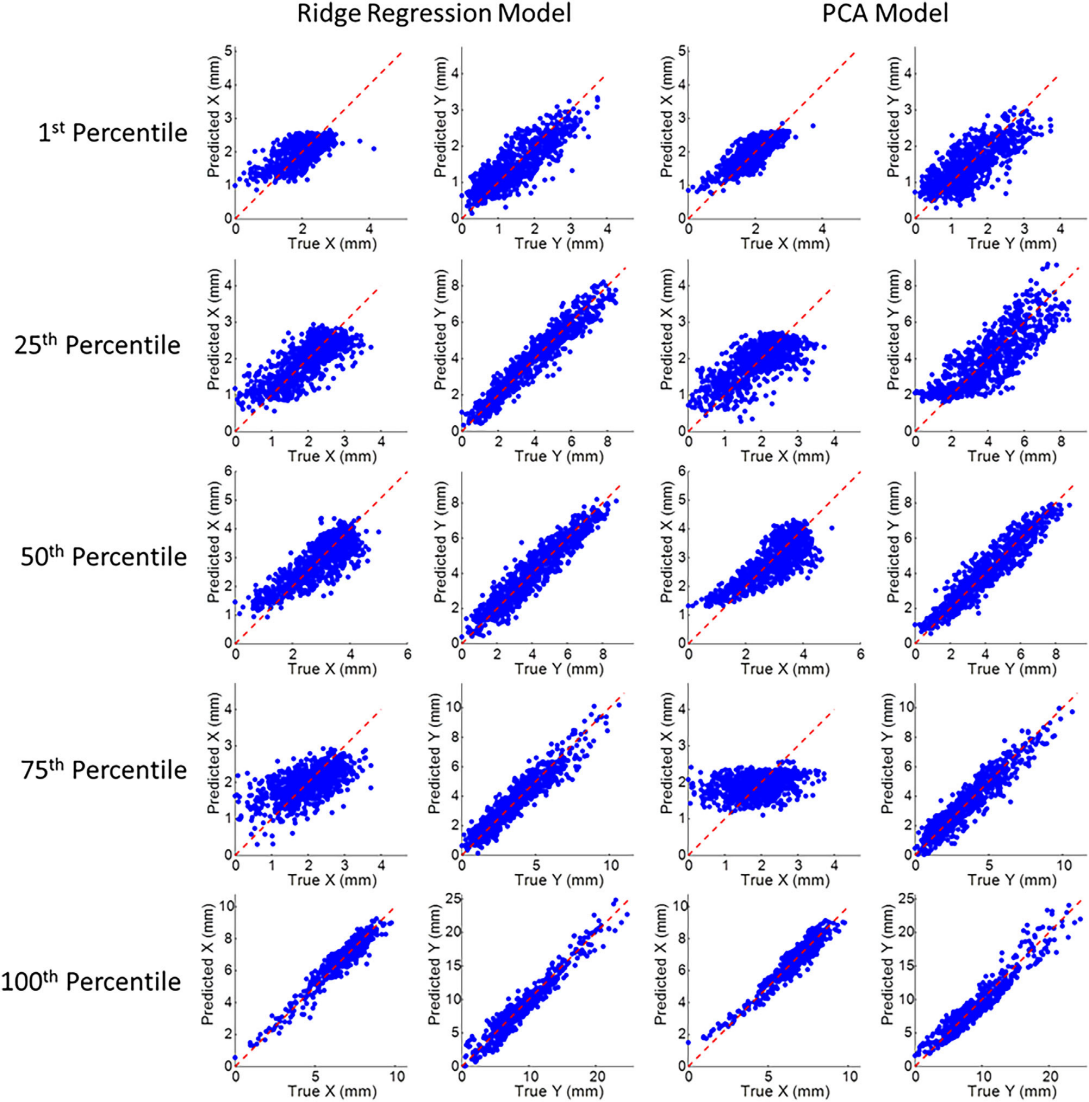
Comparison of true and predicted tumor position from diaphragm surrogate based intra‐fraction models using the ridge regression and principal component analysis methods, in the X or anterior‐posterior (AP) and the Y or superior‐inferior (SI) directions. The dashed red line represents unity. Each row corresponds to the five patients representing the 1^st^, 25^th^, 50^th^, 75^th^, and 100^th^ percentile tumor motion range in the SI direction

Table 1 displays the MSE and MAE of intra‐fraction pancreatic tumor position prediction using only the diaphragm contour surrogate. The p95 MSE was 1.0 mm^2^ (max.: 5.8 mm^2^) and 3.1 mm^2^ (max.: 17.5 mm^2^) in the AP and SI directions for the ridge regression model, while the PCA model had a p95 MSE of 1.1 mm^2^ (max.: 3.7 mm^2^) and 3.7 mm^2^ (max.: 14.0 mm^2^) in the AP and SI directions. The ridge regression model also had a p95 MAE of 0.8 mm (max.: 1.4 mm) and 1.3 mm (max.: 2.3 mm) in the AP and SI directions, with the PCA model having values of 0.8 mm (max.: 1.4 mm) and 1.4 mm (max.: 2.9 mm). Figure 6 plots true versus predicted tumor position for the same five patients.

#### 3.2.3 Tumor position prediction error using two surrogates

**FIGURE 7:**
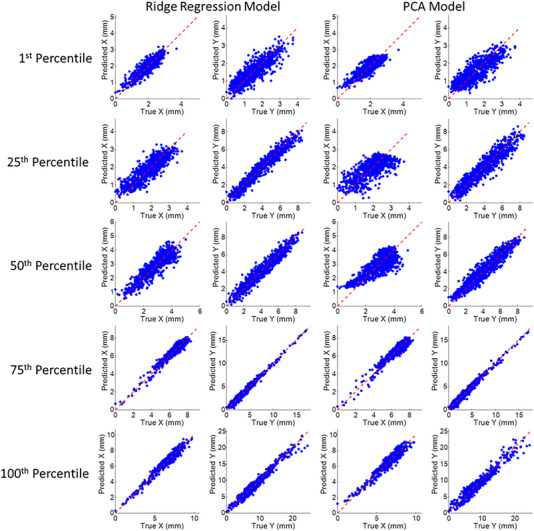
Comparison of true and predicted tumor position from abdomen and diaphragm surrogate based intra‐fraction models using the ridge regression and principal component analysis methods, in the X or anterior‐posterior (AP) and the Y or superior‐inferior (SI) directions. The dashed red line represents unity. Each row corresponds to the five patients representing the 1^st^, 25^th^, 50^th^, 75^th^, and 100^th^ percentile tumor motion range in the SI direction

The MSE and MAE of using both the abdomen and diaphragm contour surrogates to predict intra‐fraction pancreatic tumor position are shown in Table 1. Over all patients, the ridge regression model had a p95 intra‐fraction MSE of less than 1.5 mm^2^ (max.: 11.7 mm^2^) and 4.6 mm^2^ (max.: 76.78 mm^2^) in the AP and SI directions, respectively, and the p95 MAE was less than 0.9 mm (max.: 2.2 mm) and 1.8 mm (max.: 1.8 mm), in the AP and SI directions, respectively. For the PCA model, p95 intra‐fraction MSE was less than 1.7 mm^2^ (max.: 5.6 mm^2^) and 4.9 mm^2^ (max.: 13.3 mm^2^) in the AP and SI directions, and the p95 MAE was less than 1.0 mm (max.: 2.1 mm) and 1.7 mm (max.: 2.9 mm) in the AP and SI directions. Figure 7 plots true versus predicted tumor position for the same five patients.

The two surrogate model was compared using the in‐house and clinical contours for five non‐sequential patients and showed the mean square error differed by an average of 0.01 mm^2^ in the AP direction, and 0.05 mm^2^ in the SI direction.

#### 3.2.4 Full fraction tumor position prediction error

For the five patients displayed above, which demonstrated the 1^st^, 25^th^, 50^th^, 75^th^, and 100^th^ percentile tumor motion range in the SI direction, motion models were generated using 700 training frames and applied to all acquired frames. The MSE in the AP and SI motion directions for models based on the abdominal, diaphragm, or combined contour positions are shown in Table 2.

**TABLE 2:** The mean squared error (MSE) for intra‐fraction tumor position prediction models using 700 training images and applied to the entirety of the remaining frames in the anterior‐posterior (AP) and superior‐inferior (SI) directions, with the clinical tumor contour

		MSE AP (mm) Ridge		MSE SI (mm) PCA	
		Ridge	PCA	Ridge	PCA
1^st^ percentile	Abd	0.5	0.5	5.6	5.7
	Dia	4.8	0.5	2.3	5.7
	Abd+Dia	3.0	4.8	0.8	1.8
25^th^ percentile	Abd	1.3	1.3	4.2	4.2
	Dia	0.5	1.3	1.8	4.2
	Abd+Dia	1.1	1.1	4.5	4.7
50^th^ percentile	Abd	2.2	2.2	3.5	3.6
	Dia	3.0	2.2	5.5	3.6
	Abd+Dia	5.6	6.1	3.9	4.6
75^th^ percentile	Abd	0.5	0.5	1.6	1.6
	Dia	0.7	0.8	0.9	1.0
	Abd+Dia	0.5	0.5	0.8	1.0
100^th^ percentile	Abd	1.9	1.9	7.6	7.6
	Dia	4.5	1.9	4.0	7.6
	Abd+Dia	2.3	2.4	2.9	3.1

Abbreviations: Abd, abdominal contour‐based model; Dia, diaphragm contour‐based model; Abd+Dia, abdominal and diaphragm contour‐based model.

## 4 DISCUSSION

Real‐time tumor tracking during MRgRT for pancreatic cancer provides a valuable tool for accurately delivering treatment where MR‐Linac systems are available. However, due to the high cost of MR‐Linac systems, the required infrastructure, and the clinical team required to support such systems, they are not easily accessible to the majority of radiation oncology clinics. This work utilized a unique dataset obtained from our institutional MR‐Linac and treatment database to generate patient specific intra‐fraction tumor position evaluation and corresponding prediction models, using motion information from common pancreatic tumor surrogates. Motion analysis of 225 treatment fractions showed a large variation in the extent of motion present in the tumor, diaphragm, and abdominal contours. The largest tumor contour displacement was 18.5 mm in the AP direction, and 34.7 mm in the SI direction, much larger than standard treatment margin expansions, emphasizing the need for image guidance and real time target tracking to deliver safe and effective treatments. The pancreatic tumor motion range was similar to previously reported values: Knybel et al. reported a mean tumor motion of 11 mm in the SI direction tracked with the Synchrony respiratory tracking system for twenty patients, and Dolde et al. found a motion range of 3.7 mm to 28.5 mm using 4D MRI scans for nine patients compared to a mean of 7.3 ± 2.6 mm and 14.7 ± 5.7 mm in the AP and SI directions, and a range of 2.8 mm – 18.5 mm in the AP direction and 4.8 mm – 34.7 mm in the SI direction.^20^, ^21^ Utilizing real‐time cine MRI allows for a more accurate assessment of tumor and other soft tissue structure motion than respiratory‐correlated 4D‐MRI which produces time averaged images and does not show the variation in position over multiple respiratory cycles. Cine MRI also has an advantage over the Synchrony system, which relies on implanted metallic fiducials and infrared markers placed on the patient surface for tracking. The large range of tumor motion highlights the need for respiratory motion management programs to be in place when treating pancreatic cancer or other abdominal malignancies to prevent geometric miss of the target and improve normal tissue sparing. However, this study did not investigate the inter‐fraction reproducibility of surrogate motion correlation with tumor motion, or compare typical surface and implanted fiducials with the true anatomical landmarks.

The motion models developed from the cine MRI images had an average intra‐fraction MSE of 0.6 mm^2^ and 1.4 mm^2^ in the AP and SI directions, respectively, for the PCA model using the abdomen and diaphragm surrogate information, less than the typical gating margin expansion of ∼5 mm used for pancreas tumors on conventional linacs, and the 3 mm tracking margin expansion used on the MRIdian system. However, the accuracy of these models is most limited by the single 2D imaging plane and would benefit greatly from orthogonal imaging planes to provide information on the left‐right (LR) tumor motion. Due to the nature of respiratory motion, the extent of pancreatic tumor motion in the LR plane is limited and is not currently tracked during treatment delivery. The motion range in the AP and SI directions for the diaphragm and pancreas were consistent with previous literature, indicating that the single 2D imaging plane did not significantly impact motion amplitude measurements.

Analysis of the tumor position prediction models showed that the abdomen surrogate model provided the smallest error values in the AP direction and the diaphragm surrogate only models provided the smallest error values in the SI direction. In contrast, the models utilizing both surrogates to train the motion models resulted in the largest error values in both the AP and SI directions, indicating that the combination is not needed to improve our final results, and that AP and SI motion predictions should be made separately. When the entire fraction was applied with 700 training images, the MSE increased. This was especially apparent for the SI direction. The average MSE for five fractions increased from 0.7 mm^2^ to 3.0 mm^2^ in the AP direction and from 1.0 mm^2^ to 3.0 mm^2^ in the SI direction for the full fraction PCA model. This may be due to changes in the respiratory pattern over the full fraction, which ranged from 12.4 to 26.4 min in length with 2.9 min of training.

This study included some limitations. First, only 2D images were available, so complete motion information was not available for analysis. Each image set contains the AP and SI motion; however, the left‐right motion could cause changes in the perceived AP or SI motion of the target due to motion in or out of the imaging plane. Utilizing 2D cine MRI for target tracking is standard clinical practice for the ViewRay sysem, and lateral motion or deformation is accounted for in target margin formulas. Large lateral motion extent is often visible in sagittal images due to significant changes in target size or shape, and such changes were not observed for images within this dataset.^17^, ^22^, ^23^, ^24^ Second, cine MRI‐based motion and position values were not compared to conventional surface or implanted fiducial methods for target tracking. This limitation is due to the nature of MR‐Linac based treatment setup which prevents optical surface tracking and the patient population not having implanted fiducials for MRgRT. This study also relied on the ViewRay generated gating target contours for tumor centroid position, which were based on manually modified physician of the day contours using the daily pre‐treatment anatomical images. It is possible that this manual modification introduced from inter‐ and intra‐observer variation in contour shape over the five treatment fractions. The system generated gating contours were not evaluated against physician contours because the ViewRay software suite is FDA approved and the generated contours are directly utilized in clinical workflow. Additionally, the developed models were not sufficient for inter‐fraction motion prediction. Prediction error increased significantly when applying the models to inter‐fraction motion. The motion data produced in this study could also be useful for evaluating the dosimetric impact of target motion for these patients. However, this evaluation was outside the scope of the current study and will be investigated in future work.

Future works will include the addition of inter‐fraction models based on the experience and data generated from this study. Inter‐fraction models are expected to have worse prediction error and require more complex model definitions due to the variation in respiratory motion over time, especially from day‐to‐day.^22^, ^25^ To account for inter‐fraction variation some baselining method is expected to provide a starting point for the prediction. Further model development is needed to account for such variation, and our future work looks to expand the model complexity and implement machine learning techniques. Another solution to inter‐fraction variation would be the implementation of visual guidance for respiration, such as the system developed by Lewis et al. to limit the extent of motion and create a more repeatable setup.^26^

## 5 CONCLUSION

This study evaluated pancreatic tumor motion and developed patient specific intra‐fraction pancreatic tumor position prediction models for 45 patients over 225 treatment fractions. The pancreatic tumors had an average motion of 7.4 ± 2.7 mm and 14.9 ± 5.8 mm in the AP and SI directions. The ridge regression model had an MSE of 0.6 ± 0.9 mm^2^ and 1.81 ± 5.4 mm^2^ in the AP and SI directions, respectively, while the PCA model had an MSE of 0.6 ± 0.67 mm^2^ and 1.4 ± 1.69 mm^2^ in the AP and SI directions when both the abdomen and diaphragm surrogates were used in conjunction. To the authors’ knowledge, this is the first motion study of its kind, utilizing a large clinical dataset of MRgRT treatment data for assessing tumor and surrogate motion during free breathing. Additionally, this approach could be extended to other abdominothoracic cancer sites, and utilize additional surrogates. We believe this is an important tool for the eventual improvement of pancreatic cancer treatment accuracy and tumor tracking abilities, especially as an important step towards inter‐fraction motion management.

## AUTHOR CONTRIBUTIONS

Benjamin Lewis contributed to data collection, analysis, and writing of the manuscript. Anamaria Guta contributed to data analysis and reviewing of the manuscript. Jaeik Shin contributed to data analysis and model generation. Zhen Ji contributed to analysis software development and application. Jin Sung Kim contributed to study design and supervision. Taeho Kim contributed to data collection, analysis, and supervision of the project. All authors discussed the results and contributed to the final manuscript.

## CONFLICT OF INTEREST STATEMENT

The Department of Radiation Oncology provided the research funding for this work to Dr. Taeho Kim. Washington University in St. Louis has a master research agreement and receives research funding from ViewRay unrelated to this study.

## Data Availability

The data that support the findings of this study are available from the corresponding author upon reasonable request.
